# Purity of Drinking Water

**Published:** 1886-10

**Authors:** 


### Purity of Drinking Water.

The *Chemist and Druggist* says it is often required to give a quick indication of the freedom, or otherwise, of water from organic products. The rough and ready permanganate test cannot be relied upon. Most organic bodies, that is those containing nitrogen, are converted into ammonia, which is ultimately oxidized into nitrous and nitric acids. The detection of nitrous acid, therefore, is important, since its presence is sufficient to condemn any water for domestic purposes. Mr. C. C. Howard has suggested a ready test, which is as follows: Into a test-glass place some of the water (not more than 50 c.c. or ℥iss), and add a drop of hydrochloric acid, then a drop of sulphuric acid, and one of a solution of naphthylamine hydrochloride. If the water does not contain more than 1 in 100,000,000, after standing for ten minutes it should not show more than the faintest tint of pink color.

### A Fungus Developed in the Human Saliva.

M. Galippe made known the following facts at a recent meeting of the Paris Academy of Medicine. After having purified saliva by means of Pasteur's filter, M. Galippe observed, at the lower extremity of the filter, which was not in contact with the fluid, a fungus composed of tubes and spores of mycelium. Following the advice of Professor Cornu, M. Galippe cultivated this fungus in the cells of Van Tieghem, and observed that the fungus was neither an *Aspergillus* nor a *Penicillium.* This fungus, which had neither been described nor represented, belongs to the Moniliæ family. M. Galippe proposed to give it the name of *Moniliœ sputicola.* M. Charcot repeated these statements at the Academy of Sciences, in the name of M. Galippe.

